# 

**DOI:** 10.4103/0974-7788.72482

**Published:** 2010

**Authors:** Urmila Thatte

**Affiliations:** *Professor and Head, Department of Clinical Pharmacology, GSMC and KEM Hospital, Parel, Mumbai, India. E-mail: urmilathatte@gmail.com*

I write the editorial for this third issue with the plea that all researchers read, internalize and practice the principles enunciated in the “The Singapore Statement on Research Integrity” which was developed as part of the 2^nd^ World Conference on Research Integrity, 21–24 July 2010. The objective of this effort was to develop a “global guide to the responsible conduct of research that could serve as an international framework for responsible conduct of research, stimulate further debate, and promote the translation of core principles into more detailed guidance documents for specific issues.”[1]

Four principles have been laid down in the statement, namely, honesty in all aspects of research, accountability in the conduct of research, professional courtesy and fairness in working with others and good stewardship of research on behalf of others.[2] These are not new principles and perhaps only re-emphasize all aspects of ethical research enshrined in the Declaration of Helsinki.[3]

The statement further highlights 14 responsibilities of researchers, including integrity where it is expected that a researcher take responsibility for the trustworthiness of his/her research. This is so important because increasingly in the recent past we have seen cases where major research papers that had great potential in changing clinical practice have been withdrawn due to fraud. Take the recent example of the retraction of two papers (one of which has been cited more than 190 times) on the mechanism of estrogen signaling. This happened after the pharmaceutical company Wyeth (now Pfizer) found that its former employee Boris Cheskis had published these papers based on “unreliable” data.[4]

When falsified data are published (and history is dotted with many more examples), this can lead to inappropriate clinical applications, and also newer research based on it which results in further waste of time and resources. Research integrity of individual researchers is important; more so is the integrity of journals. It is shocking to read reports of journals published by reputed publishing houses that looked like peer reviewed medical journals, but were sponsored by unnamed pharmaceutical companies.[5]

The Singapore Statement further adds the need for adherence to regulations by researchers. Adherence to ethical guidelines is also needed and investigators cannot get away saying they “were not aware” of regulations/ethical guidelines. Similarly, need for using appropriate research methods, keeping auditable research records, and sharing research findings (by publication) are other responsibilities mentioned in the statement. These are reiterations of the sentiments expressed in the Declaration of Helsinki. Authorship and acknowledgment issues need to be addressed by researchers. The problems that beset researchers while deciding authorship must be addressed at the beginning of the research rather than when the manuscript is being written and the International Committee of Medical Journal Editors has issued excellent guidelines about who should be an author, for all to follow.[6]

The need for peer review is important. However, the need for researchers to respect confidentiality when reviewing others’ work has been highlighted in the statement. This statement directly leads to the next responsibility – that of declaring conflict of interest. Another core ethical principle, it is important that both researchers and peer reviewers should disclose any conflicts of interest that could compromise their work.

Another responsibility described relates to public communication of research – this is well described in the ICMR guidelines for ethical clinical research.^7^ Reporting and responding to irresponsible research practices, the importance of an appropriate research environment (which encourages research integrity) and societal considerations form the rest of the responsibilities described.
